# The First Complete Mitogenome Characterization of Brown Alga *Dictyota coriacea* (Phaeophyceae, Heterokontophyta) and Its Phylogenetic Analysis

**DOI:** 10.3390/life15101605

**Published:** 2025-10-15

**Authors:** Maheshkumar Prakash Patil, Hee-Eun Woo, Young Jae Jeon, Shin-Ichi Kitamura, Young-Ryun Kim, Jong-Oh Kim, Kyunghoi Kim

**Affiliations:** 1 Industry-University Cooperation Foundation, Pukyong National University, 45 Yongso-ro, Nam-Gu, Busan 48513, Republic of Korea; 2 Department of Microbiology, Pukyong National University, 45 Yongso-ro, Nam-Gu, Busan 48513, Republic of Korea; 3 Institute of Sustainable Earth and Environmental Dynamics (SEED), Pukyong National University, 45 Yongso-ro, Nam-Gu, Busan 48513, Republic of Korea; 4 Graduate School and Faculty of Bioresources, Mie University, 1577 Kurimachiya-cho, Tsu City 514-8507, Mie Prefecture, Japan; 5 Marine Eco-Technology Institute, Busan 48520, Republic of Korea; 6 School of Marine and Fisheries Life Science, Pukyong National University, 45 Yongso-ro, Nam-Gu, Busan 48513, Republic of Korea; 7 Department of Ocean Engineering, Pukyong National University, 45 Yongso-ro, Nam-Gu, Busan 48513, Republic of Korea

**Keywords:** *Dictyota coriacea*, mitochondrial genome, marine algae, Phaeophyceae, phylogenetic analysis, gene structure, codon usage

## Abstract

Brown algae (Phaeophyceae) play vital ecological roles in marine ecosystems and are important models for studying organelle genome evolution. Despite their significance, mitogenome data for many taxa remain limited. In this study, we present the complete mitogenome sequence of *Dictyota coriacea*, a representative brown alga from the family Dictyotaceae (Phaeophyceae). The circular mitogenome of *D. coriacea* is 31,573 bp in length and encodes 62 genes, including 35 protein-coding genes (PCGs; including uncharacterized open reading frame (*orf109*)), 25 tRNAs, and 3 rRNAs. The overall gene content and arrangement are largely conserved and consistent with other Dictyotaceae species. However, minor but notable genomic variations were observed, such as gene overlaps, variation in gene lengths, and differences in tRNA gene copy numbers, and the absence of *rpl31*. All PCGs use standard start and stop codons, with most initiating with ATG and terminating with TAA, TAG, or TGA. Phylogenetic analysis confirmed *D. coriacea’s* close relationship with *D. dichotoma*, *Dictyopteris divaricata*, and *Dictyotopsis propagulifera*, supporting its taxonomic placement. This study’s findings improve our understanding of algae evolution and provide useful genetic markers for future research on evolutionary relationships and species classification within this group of algae.

## 1. Introduction

Brown algae (Phaeophyceae) represent an ecologically and economically significant group of marine macroalgae that dominate temperate and tropical coastal environments. They contribute significantly to primary production, provide habitats for a wide range of marine organisms, and play a key role in nutrient cycling [1,2]. Among them, the genus *Dictyota* J.V. Lamouroux, belonging to the order Dictyotales, includes species commonly found in intertidal and subtidal zones. These algae are recognized not only for their ecological roles but also for their capacity to produce a variety of bioactive secondary metabolites with potential applications in biotechnology [3,4,5]. *Dictyota coriacea* is one such brown algal species distributed in coastal habitats. Its thallus is tough, flattened, dichotomously branched, and well adapted to withstand dynamic marine environments [5,6]. Despite the ecological and economic importance of Dictyotaceae, complete mitochondrial genomes within this family remain limited, creating gaps in our understanding of their evolutionary dynamics. Sequencing and characterizing the mitogenome of *D. coriacea* fills a critical gap by providing novel genomic data that strengthens phylogenetic resolution within Dictyotales and the broader Phaeophyceae lineage. This information not only clarifies taxonomic relationships but also supports broader applications in comparative genomics, evolutionary studies, and marine biotechnology, where mitochondrial markers are increasingly used for species identification, population genetics, and bioprospecting of bioactive compounds.

Mitogenomes have become a valuable resource for phylogenetic and evolutionary studies due to their compact size, conserved gene content, and relatively low recombination rates [7]. In brown algae, mitogenomes are typically circular and contain genes involved in respiration, energy production, and protein synthesis [8,9]. Unlike morphological traits, which can be highly plastic and environmentally influenced, mitogenomic features offer stable and reliable markers for evolutionary analysis [10]. The use of complete mitogenomes in phylogenetics provides a much higher resolution compared to single or partial gene analyses because they encompass the full set of PCGs, rRNAs, and tRNAs, thereby offering a larger number of informative sites. This reduces lineage-specific biases often associated with single-gene phylogenies, producing more robust and well-supported evolutionary relationships. Several studies have demonstrated that mitogenome-based phylogenies can resolve relationships not only among families and genera but also among closely related species of brown algae [8,11,12,13,14]. This is particularly valuable in algal taxonomy, where morphological convergence and phenotypic plasticity can obscure true evolutionary relationships [14,15]. Furthermore, mitogenomic data can aid in identifying cryptic species, uncovering gene rearrangements, and detecting horizontal gene transfer events [10]. As highlighted by Bringloe et al. [14], organelle phylogenomics, including complete mitogenome datasets, provides finer-scale resolution within brown algae and has significantly improved our understanding of diversification and taxonomic relationships within the Phaeophyceae.

Although many brown algal mitogenomes have been sequenced over the last decade, there remains a significant gap in genomic data for several lineages, including the genus *Dictyota*. The lack of complete mitogenome sequences hinders comprehensive comparative and evolutionary studies. Therefore, sequencing the mitogenome of *D. coriacea* contributes to filling this gap and provides a reference for future research on Dictyotales. In addition to expanding genetic resources, mitogenome studies can reveal novel structural features such as intron patterns, gene duplications, and tandem repeats that shed light on the dynamics of genome evolution [9,16]. Features like codon usage bias, strand asymmetry, and nucleotide composition can also provide clues about the organism’s adaptation to its environment and evolutionary constraints.

In this study, we report the complete mitogenome of *Dictyota coriacea* collected from the shoreline of Busan, South Korea. We describe its genome organization, gene content, and base composition, and compare it with other known brown algal mitogenomes. Additionally, we conduct a comprehensive phylogenetic analysis using complete mitogenome sequences from 53 Phaeophycean species to determine the evolutionary position of *D. coriacea*. This study represents the first complete mitogenome report for this species and contributes to a broader understanding of mitogenome evolution in brown algae. The findings are expected to support future efforts in taxonomy, molecular ecology, and marine biotechnology.

## 2. Materials and Methods

### 2.1. Sample Collection, DNA Extraction, and Sequencing

In March 2024, a specimen of the brown macroalga *Dictyota coriacea* (Figure S1) was collected from the coastal region of Busan, Korea (35°16′ N, 129°15′ E). The sample was deposited at the Marine Eco-Technology Institute’s Ecological Restoration Group in Busan, where it was cataloged under voucher number PU-T01-S-MA-09 (contact: Dr. Young-Ryun Kim, yykim@marineeco.co.kr). Genomic DNA was isolated using the DNeasy Plant Kit (Qiagen, Hilden, Germany), following the standard protocol provided by the manufacturer. DNA quantification was carried out using a NanoDrop spectrophotometer (Thermo Fisher Scientific D1000, Waltham, MA, USA), and the extracted DNA was preserved at −20 °C until further use. For library preparation and sequencing, the genomic DNA was prepared using the TruSeq Nano DNA Library Preparation Kit (Illumina, San Diego, CA, USA) and sequenced on the Illumina HiSeq platform at Macrogen (Daejeon, South Korea; https://www.macrogen.com/ko/) using the Illumina HiSeq 2500 platform (Illumina, San Diego, CA, USA), with paired-end reads (2 × 150 bp) and an average insert size of 350 bp.

### 2.2. Sequence Assembly, Annotation and Analysis

Low-quality reads (Q < 20) and adapter sequences were removed using Trimmomatic v0.36 to improve data accuracy [17]. A random set of the cleaned reads was selected for de novo assembly of the mitogenome. FastQC v0.11.5 was used to check the quality of the sequencing data [18]. The de novo assembly of the *D. coriacea* mitogenome was carried out using NOVOPlasty v4.2.1 [19], based on Illumina short-read data. *Dictyota dichotoma* (AY500368) mitogenome provided as the seed and reference sequence. The assembly produced a high-quality circular mitogenome with consistent coverage and read depth. The detailed assembly statistics, including read quality, coverage, and assembly parameters, are summarized in Table S1. The resulting sequences were compared with known mitogenomes in the NCBI database using BLAST. Genome annotation was performed with MFannot [20], using Translation Table 1 (standard). Protein-coding genes (PCGs) were identified with ORF Finder and confirmed by BLAST searches against the NCBI protein database [21]. tRNA genes were predicted using tRNAscan-SE 2.0 [22], and RNAweasel helped confirm RNA locations and detect introns [23]. Tandem Repeats Finder v4.09 was used to identify repeated sequences [24]. A circular genome map was created with OGDRAW v1.3.1 [25]. MEGA11 v11.2.8 was used to calculate nucleotide composition and codon usage patterns (RSCU) [26]. Strand asymmetry was measured using the formulas: GC-skew = (G − C)/(G + C) and AT-skew = (A − T)/(A + T) [27].

### 2.3. Phylogenetic Analysis

To determine the evolutionary placement of *D. coriacea* within the brown algae (Phaeophyceae), complete mitochondrial genome sequences from 54 species available in GenBank were compiled. Of these, 53 species were included as the ingroup, with *Schizocladia ischiensis* designated as the outgroup (Table S2). The phylogenetic relationships within Phaeophyceae were inferred using 35 PCGs, comprising 17 ribosomal proteins (*rps2–4*, *7*, *8*, *10*–*14*, and *19*; *rpl2*, *5*, *6*, *14*, *16*, *31*), ten NADH dehydrogenase subunits (*nad1*–*7*, *4L*, *9*, *11*), three ATPase subunits (*atp6*, *8*, *9*), three cytochrome oxidase subunits (*cox1*–*3*), a secY-independent transporter protein (*tatC*), and apocytochrome b (*cob*). Multiple sequence alignment was performed using the online version of MAFFT v7.0 [28]. Then, maximum likelihood (ML) and Bayesian Inference (BI) methods were performed. The best-fit model for tree construction was selected using Modelfinder in IQ-TREE, based on the Bayesian information criterion (BIC) [29]. A phylogenetic tree was then built using the ML method with the GTR+F+I+G4 model in IQ-TREE [30]. To assess the robustness of the inferred topology, ultrafast bootstrap support values were calculated from 1000 replicates. BI phylogenetic analysis was conducted using MrBayes version 3.1.2, applying the GTR+I model (nst = 6) with one cold chain and three heated Metropolis-coupled Markov chain Monte Carlo (MCMCMC) chains. The analysis ran for 10 million generations, sampling every 100 generations, with the initial 25% of samples discarded as burn-in. The resulting tree was visualized using the iTOL v.7 web tool [31].

## 3. Results and Discussion

### 3.1. Mitogenome Structure and Nucleotide Composition

**Figure 1:**
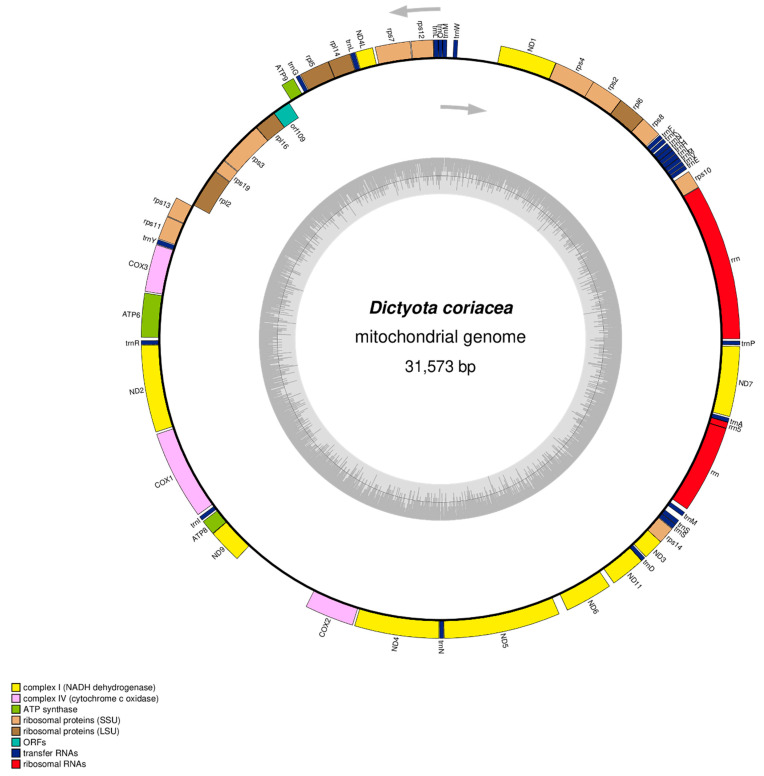
The circular mitogenome of *Dictyota coriacea* (GenBank accession number: PV670818). The arrow directions show gene orientation, the different colors reflect the grouping of functional genes together with their acronyms, and the inner circle indicates the GC contents.

The complete mitogenome of *D. coriacea* was successfully sequenced and assembled. The finalized mitogenome was 31,573 bp in length and has been submitted to GenBank under the accession number PV670818. A visual representation of the gene map is provided in Figure 1, while key genome characteristics are summarized in Table 1. The mitogenome of *D. coriacea* comprises 63 genes, including 35 PCGs (including uncharacterized open reading frame (*orf109*)), 25 tRNAs, and 3 rRNAs (Table 1). These genes are responsible for critical mitochondrial functions such as protein synthesis, electron transport, ATP production, and protein translocation (Table S3). Interestingly, only five PCGs (*tatC*, *rpl16*, *rps3*, *rps19*, *rpl2*) and *orf109* gene are located on the light (L) strand, with the remaining genes situated on the heavy (H) strand, which is consistent with the strand bias observed in other brown algal mitogenomes [12,32,33]. Intergenic region analysis revealed a total of nine overlapping regions between adjacent genes ranging from 1 to 12 bp, collectively spanning 58 bp. In contrast, the total non-coding interval between adjacent genes was 1457 bp, accounting for approximately 4.61% of the entire mitogenome length. This balance between coding and non-coding regions suggests a compact genome structure with minimal non-functional sequence space. The overall nucleotide composition of the *D. coriacea* mitogenome was 25.4% adenine (A), 35.8% thymine (T), 22.4% guanine (G), and 14.9% cytosine (C), resulting in a total AT content of 61.2% (Table 2). Compared to other members of the Dictyotaceae family, *D. coriacea* showed slightly lower AT content and marginally higher GC content. The mitogenome exhibited a negative AT skew and a positive GC skew, indicating a higher frequency of thymine over adenine and guanine over cytosine, a trend commonly observed in mitogenomes of brown algae [12,32,33,34,35].

Comparative analysis with other Dictyotaceae species revealed both conserved and unique features in the *D. coriacea* mitogenome (Table 2). Most mitogenomes in this family range between 30 and 32 kb in length and typically encode 34–35 PCGs, 23–25 tRNAs, and 3 rRNAs, all within a similarly biased AT-rich context [12,32,33]. The *D. coriacea* mitogenome closely aligns with this pattern in terms of gene content and organization, supporting its phylogenetic placement within family Dictyotaceae. Further comparison indicated that *D. coriacea* shares higher sequence similarity with congeneric species such as *D. dichotoma* and *Dictyopteris* species. This similarity extends to gene order (synteny), gene content, and conserved functional regions, suggesting strong evolutionary conservation within the group. Notably, the core PCGs identified in *D. coriacea* encode essential subunits of the oxidative phosphorylation pathway, including NADH dehydrogenase, cytochrome oxidase, and ATP synthase complexes. These proteins play central roles in energy metabolism, and their conservation across species to maintain their structure and function. The presence of a full complement of tRNA genes further emphasizes the functional completeness of the mitochondrial translation machinery in *D. coriacea*. However, despite overall conservation, notable differences in intergenic regions and the presence of unique ORFs (such as *orf109*) point to genomic rearrangements or adaptations. These variations may represent evolutionary responses to distinct environmental conditions experienced by *D. coriacea*, such as temperature, salinity, or habitat specialization [12,15].

### 3.2. Protein-Coding Gene Features

The mitogenome of *D. coriacea* encodes a total of 35 PCGs, collectively spanning 23,928 bp, which accounts for approximately 75.96% of the entire mitogenome (Table 1). Among these, *nad5* was identified as the longest gene (1992 bp), while *atp9* was the shortest (228 bp). The overall nucleotide composition and base skew values for each PCG are summarized in Table 3. The average AT content across all PCGs was 61.8%, with individual genes ranging from 56.8% (*nad7*) to 69.3% (*atp8*). Furthermore, base skew analysis revealed that eight PCGs exhibited positive AT-skew, and 31 PCGs displayed positive GC-skew, suggesting strand-specific mutational biases and replication–transcription asymmetries influencing nucleotide composition. Such skew patterns are well-documented in organellar genomes and are often associated with underlying genomic architecture and functional constraints [13,36,37]. Within the Dictyotaceae family, most species possess 34 to 35 PCGs, showing high conservation of mitochondrial gene content. Notably, *D. coriacea*, along with *D. dichotoma* and *D. divaricata*, contains an additional hypothetical ORFs (Table 2). However, *D. coriacea* differs by the absence of the *rpl31* gene, a ribosomal protein gene that is present in some related species (Table 4) [12,32,33]. While most PCGs are conserved across the family, length variation was observed in 26 of the 34 PCGs among *D. coriacea* and three other Dictyotaceae family species, indicating that although gene content is largely stable, sequence-level changes reflect evolutionary divergence (Table 4). These variations in gene length and base composition are likely driven by a combination of strand asymmetry, environmental adaptation, and evolutionary streamlining, all of which are known factors influencing mitogenome evolution in algae [9,15,36,38]. Regarding translation initiation, ATG was identified as the standard start codon for all PCGs in *D. coriacea*, except for *rps14*, which initiates with GTG-a variation also observed in *Dictyota dichotoma*, while in contrast, *Dictyopteris divaricata* and *Dictyotopsis propagulifera* use ATG for the same gene. This codon variation reflects specific translational preferences within family Dictyotaceae (Table 4) [12]. In terms of translation termination, most PCGs in *D. coriacea* end with the canonical TAA stop codon. However, exceptions include TAG (in *rps10*, *rps4*, *rps12*, and *rpl5*) and TGA (in *rps8*, *rpl14*, *rpl16*, *atp8*, *cob*, and *nad11*). When compared with other family members, *D. coriacea* shares 14 of 34 PCG stop codons with other species, while 20 genes show distinct termination patterns, highlighting moderate codon variability within the family. Overall, the PCGs structure, organization, and content in the *D. coriacea* mitogenome are highly consistent with those of other Dictyotaceae members, underscoring a conserved mitogenomic architecture at the family level. These findings also align with previous reports that mitogenomes in brown algae generally maintain order-level conservation across the class Phaeophyceae [8,12,13]. However, recent mitogenomic studies have increasingly revealed that structural diversity exists both within and between taxonomic levels, including inter- and intra-order variability across Phaeophycean lineages [9,12]. This underscores the evolutionary dynamics of algal mitochondria, where core metabolic genes remain conserved, while non-coding regions, gene lengths, and specific codon usage may diverge due to adaptive requirements.

The codon usage and RSCU analysis of the mitochondrial PCGs of D. coriacea are summarized in Table S4. The total length of PCGs was 23,982 bp, encoding 7959 codons (excluding stops), corresponding to 61 sense codons for 20 standard amino acids. Among these, UUU (phenylalanine) was the most frequent codon (512 times), while UUA (leucine) showed the highest RSCU value (2.68), indicating strong codon preference. In contrast, AUG (methionine), UGG (tryptophan), and GAA/GAG (glutamate) exhibited no bias (RSCU = 1). Overall, 25 codons had RSCU > 1, suggesting preferential use, while 32 had values < 1 [39]. These trends are largely consistent with other Dictyotaceae species, such as *Dictyota dichotoma*, *Dictyopteris divaricata*, and *Dictyopteris propagulifera* (Table S4), supporting evolutionary conservation in codon usage within the family. Subtle differences likely reflect the combined effects of mutation pressure, natural selection, and genomic constraints [9,12,15,36]. Codon bias plays an important role in translational efficiency by aligning codon choice with available tRNA pools, optimizing protein synthesis. In algal mitogenomes, this bias may also be shaped by compact genome structure, high AT content, and reduced recombination rates [9,15]. While this study provides baseline insights into codon usage in *D. coriacea*, broader comparative analyses integrating transcriptomic and proteomic data will be necessary to fully assess the functional and adaptive implications of these patterns.

### 3.3. Ribosomal and Transfer RNA Genes

The mitogenome of *D. coriacea* exhibits both conserved and distinct features in its RNA gene composition relative to other Dictyotaceae members. It contains three rRNA genes-*rnl*, *rns*, and *rrn5*—all located on the heavy (H) strand, consistent with the typical mitogenome structure observed in brown algae of this family (Table S5). A notable structural characteristic is the overlap between *rrn5* and *rns* by 11 nucleotides, and between *rrn5* and *trnA* by 6 nucleotides (Table 1), indicating a compact gene arrangement. Compared to species such as *Dictyota dichotoma*, *Dictyopteris divaricata*, and *Dictyopteris propagulifera*, *D. coriacea* features a longer *rnl* and shorter *rns* and *rrn5* genes (except for *rns* in *D. propagulifera*) [12,32,33]. These variations suggest species-specific structural adaptations likely driven by evolutionary divergence [9,15]. Differences in rRNA gene length may influence ribosome assembly and translational efficiency through altered RNA folding and protein interactions [40], reflecting lineage-specific adaptations to mitochondrial function and environmental pressures [41]. The observed gene overlaps further emphasize mitogenome compactness, likely shaped by selection for efficient replication and transcription—an evolutionary trend common in algal organelles [8,12,13].

In addition to rRNA genes, the mitogenome of *D. coriacea* includes 25 tRNA genes, which collectively span 1886 bp and represent approximately 5.97% of the entire mitogenome. The length of individual tRNA genes ranges from 72 to 86 bp (Table 1), reflecting the inherent structural diversity of these essential RNA molecules. Within the Dictyotaceae family, the number of tRNA genes varies slightly-from 23 to 25-across different species (Table S6), which is consistent with the observed variation in *D. coriacea*. Characteristic features of *D. coriacea*’s tRNA profile include the presence of three *trnL* (CAA, TAA, TAG) genes, two *trnS* (GCT, TGA) genes, and multiple copies of *trnM* (CAT). These patterns are similar to those observed in other Dictyotaceae species and suggest a functional redundancy or potential regulatory roles for certain tRNAs. The duplication and retention of multiple tRNA copies in mitogenomes could be a strategy to support efficient translation or mitigate the effects of gene loss or mutation over evolutionary time [9]. Although the overall tRNA and rRNA gene arrangement appears largely conserved across Dictyotaceae mitogenomes, the presence or absence of certain genes and differences in gene copy number indicate ongoing evolutionary fine-tuning. Such genomic flexibility likely related to energy metabolism, organelle function, and environmental adaptation [12,15].

### 3.4. Phylogenetic Relationship Within Phaeophyceae

**Figure 2:**
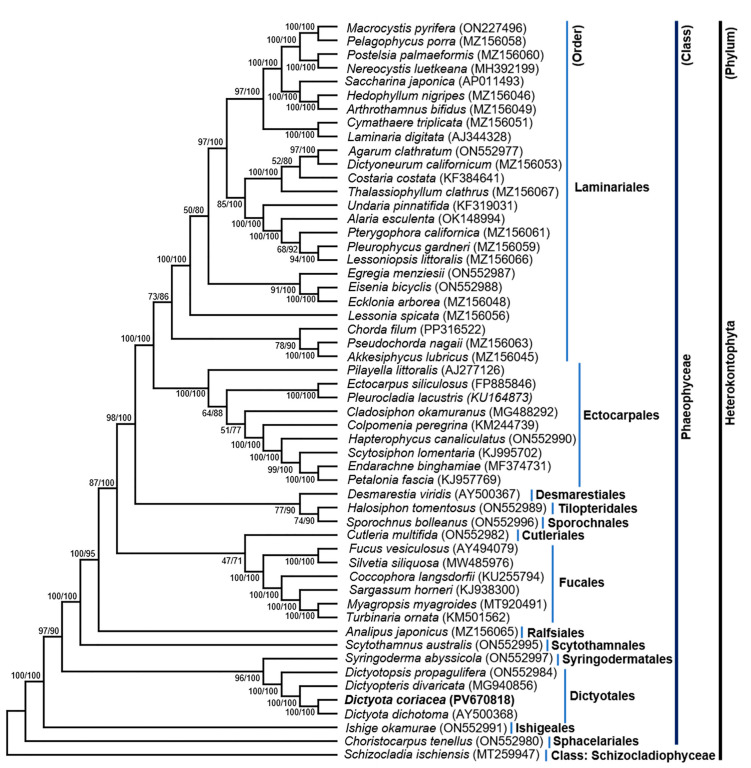
Phylogenetic tree constructed using 35 PCGs of mitogenome sequences of taxa within Phaeophyceae. The tree was rooted with *Schizocladia ischiensis* (Schizocladiophyceae). The numbers at internal nodes (ML/BI) indicate Maximum Likelihood (ML) bootstrap values and Bayesian Inference (BI) posterior probability values, respectively. The tree efficiently distinguishes *Dictyota coriacea* (in this study) from other members of the class Phaeophyceae.

The ML/BI phylogenetic tree based on 35 PCGs sequences of representative Phaeophyceae species revealed a well-resolved topology with strong bootstrap support at most nodes (Figure 2). *D. coriacea*, the focal species of this study, was recovered in a strongly supported clade within the order Dictyotales, showing 100% bootstrap values at most major nodes. Within Dictyotaceae, *D. coriacea* clustered closely with its congener *D. dichotoma* and formed a sister clade with *Dictyotopsis divaricata* and *Dictyotopsis propagulifera*. This placement confirms its taxonomic assignment to the genus *Dictyota* and underscores its close evolutionary relationships with other Dictyotaceae members, consistent with previous phylogenies based on mitochondrial and plastid genomes [10,12]. The close evolutionary relationships among these species likely reflect conserved mitochondrial gene architecture, including similar PCG content, gene arrangement, and codon usage bias, as observed in comparative mitogenomic analyses. Such patterns have been previously reported in other brown algal taxa as well, highlighting the role of mitogenome evolution in species divergence within Phaeophyceae [9,12].

Moreover, the long branch length leading to *D. coriacea* suggests that mitogenome divergence has occurred, possibly due to species-specific evolutionary pressures or geographic isolation. This is consistent with unique genomic features observed in *D. coriacea*, such as gene overlaps and codon usage bias. Similar cases in other algae suggest that such branch elongation may reflect lineage-specific rate heterogeneity or incomplete taxon sampling [16]. Since few Dictyotales mitogenomes are currently available, under sampling may exaggerate this effect, highlighting the need for broader taxon coverage. The tree also clearly separates major Phaeophyceae families, including Dictyotaceae, Fucaceae, Sargassaceae, and Laminariales, supporting deep evolutionary divergences consistent with recent mitogenome-based reconstructions [8,13,42]. As emphasized by Bringloe et al. [14], complete mitogenomes provide far better phylogenetic resolution than single-gene datasets, underscoring the importance of expanded sampling for understanding diversification and adaptation in brown algae. Overall, the phylogeny confirms the close relationship of *D. coriacea* with *D. dichotoma* and validates its placement within Dictyotaceae.

## 4. Conclusions

The complete mitogenome of *D. coriacea* reveals a largely conserved gene structure and organization typical of the Dictyotaceae family, while also exhibiting minor but meaningful variations such as gene overlaps, differences in rRNA and tRNA gene lengths, and variability in tRNA copy numbers. The conserved features of PCGs and mitochondrial RNA genes in *D. coriacea* are consistent with patterns seen across Phaeophyceae, while also indicating slight functional or adaptive variations specific to this species. Phylogenetic analysis based on complete mitogenome sequences further confirmed the close evolutionary relationship of *D. coriacea* with its congeneric species (*D. dichotoma*), and other family members (*D. propagulifera* and *D. divaricata*), firmly placing it within a well-supported clade of Dictyotaceae and affirming its taxonomic position. Altogether, these findings contribute valuable insights into the mitogenomic evolution of brown algae and underscore the importance of mitochondrial data in resolving phylogenetic relationships. This newly sequenced mitogenome represents an important genomic resource for future research on evolutionary dynamics, biodiversity, barcoding, phylogeography, population genetics, and ecological adaptations within Phaeophyceae.

## Figures and Tables

**Table 1:** List of annotated genes, including their boundaries, sizes, and intergenic nucleotides (IN), start and stop codons, anticodons and number of amino acids for *Dictyota coriacea*.

Gene	Position		Size (bp)	Coding Strand	IN	Codon		Anti-Codon	Amino Acids
	Start	End				Start	Stop		
*rnl*	1	2668	2668	H	32	-	-	-	-
*rps10*	2673	2987	315	H	4	ATG	TAG	-	104
*trnE*	3037	3108	72	H	49	-	-	TTC	-
*trnV*	3115	3188	74	H	6	-	-	TAC	-
*trnM*	3196	3268	73	H	7	-	-	CAT	-
*trnL*	3271	3354	84	H	2	-	-	TAA	-
*trnH*	3360	3433	74	H	5	-	-	GTG	-
*trnC*	3436	3508	73	H	2	-	-	GCA	-
*trnN*	3512	3584	73	H	3	-	-	GTT	-
*trnK*	3608	3680	73	H	23	-	-	TTT	-
*trnF*	3694	3766	73	H	13	-	-	GAA	-
*rps8*	3783	4154	372	H	16	ATG	TGA	-	123
*rpl6*	4151	4642	492	H	−4	ATG	TAA	-	163
*rps2*	4642	5199	558	H	−1	ATG	TAA	-	185
*rps4*	5189	5902	714	H	−11	ATG	TAG	-	237
*nad1*	5904	6872	969	H	1	ATG	TAA	-	322
*tatC*	7586	6861	726	L	−12	ATG	TAA	-	241
*trnW*	7600	7672	73	H	13	-	-	CCA	-
*trnM*	7784	7856	73	H	111	-	-	CAT	-
*trnQ*	7858	7929	72	H	1	-	-	TTG	-
*trnL*	7930	8009	80	H	0	-	-	TAG	-
*rps12*	8013	8393	381	H	3	ATG	TAG	-	126
*rps7*	8404	9003	600	H	10	ATG	TAA	-	199
*nad4L*	9042	9344	303	H	38	ATG	TAA	-	100
*trnL*	9352	9433	82	H	7	-	-	CAA	-
*rpl14*	9439	9825	387	H	5	ATG	TGA	-	128
*rpl5*	9828	10,343	516	H	2	ATG	TAG	-	171
*trnG*	10,354	10,426	73	H	10	-	-	GCC	-
*atp9*	10,474	10,701	228	H	47	ATG	TAA	-	75
*orf109*	11,086	10,757	330	L	55	ATG	TAA	-	109
*rpl16*	11,490	11,083	408	L	−4	ATG	TGA	-	135
*rps3*	12,314	11,493	822	L	2	ATG	TAA	-	273
*rps19*	12,565	12,323	243	L	8	ATG	TAA	-	80
*rpl2*	13,291	12,572	720	L	6	ATG	TAA	-	239
*rps13*	13,316	13,660	345	H	24	ATG	TAA	-	114
*rps11*	13,667	14,071	405	H	6	ATG	TAA	-	134
*trnY*	14,083	14,164	82	H	11	-	-	GTA	-
*cox3*	14,172	14,984	813	H	7	ATG	TAA	-	270
*atp6*	15,008	15,760	753	H	23	ATG	TAA	-	250
*trnR*	15,811	15,883	73	H	50	-	-	TCT	-
*nad2*	15,888	17,375	1488	H	4	ATG	TAA	-	495
*cox1*	17,410	18,939	1530	H	34	ATG	TAA	-	509
*trnI*	18,980	19,051	72	H	40	-	-	GAT	-
*atp8*	19,078	19,338	261	H	26	ATG	TGA	-	86
*nad9*	19,341	19,928	588	H	2	ATG	TAA	-	195
*cob*	19,943	21,097	1155	H	14	ATG	TGA	-	384
*cox2*	21,338	22,183	846	H	240	ATG	TAA	-	281
*nad4*	22,215	23,657	1443	H	31	ATG	TAA	-	480
*trnN*	23,664	23,735	72	H	6	-	-	ATT	-
*nad5*	23,739	25,730	1992	H	3	ATG	TAA	-	663
*nad6*	25,876	26,697	822	H	145	ATG	TAA	-	273
*nad11*	26,782	27,384	603	H	84	ATG	TGA	-	200
*trnD*	27,383	27,455	73	H	−2	-	-	GTC	-
*nad3*	27,476	27,835	360	H	20	ATG	TAA	-	119
*rps14*	27,829	28,125	297	H	−7	GTG	TAA	-	98
*trnS*	28,132	28,217	86	H	6	-	-	GCT	-
*trnS*	28,221	28,305	85	H	3	-	-	TGA	-
*trnM*	28,397	28,472	76	H	91	-	-	CAT	-
*rns*	28,537	30,061	1525	H	64	-	-	-	-
*rrn5*	30,051	30,163	113	H	−11	-	-	-	-
*trnA*	30,158	30,230	73	H	−6	-	-	TGC	-
*nad7*	30,254	31,450	1197	H	23	ATG	TAA	-	399
*trnP*	31,470	31,541	72	H	19	-	-	TGG	-

**Table 2:** General features of the complete mitogenome of family Dictyotaceae species (ORF * indicates unidentified reading frame).

Species	Size (bp)	Nucleotide Composition (%)						AT-Skew	GC-Skew	Number of Genes			
		A	T	G	C	A+T	G+C			PCG	tRNA	rRNA	ORF *
*Dictyota coriacea*	31,573	25.4	35.8	22.4	16.5	61.2	38.8	−0.170	0.153	34	25	3	1
*Dictyota dichotoma*	31,617	25.9	37.5	21.6	14.9	63.5	36.5	−0.183	0.182	35	25	3	3
*Dictyopteris divaricata*	32,021	25.5	36.2	21.8	16.5	61.7	38.3	−0.174	0.139	35	24	3	3
*Dictyotopsis propagulifera*	30,995	32.4	40.7	15.3	11.5	73.2	26.8	−0.113	0.140	35	23	3	0

**Table 3:** Nucleotide composition and skewness of PCGs in the mitogenome of *Dictyota coriacea*.

PCG	Length (bp)	Nucleotide Composition (%)						AT-Skewness	GC-Skewness
		A	T	G	C	A+T	G+C		
*rps10*	315	29.8	35.6	19.7	14.9	65.4	34.6	−0.087	0.138
*rps8*	372	23.7	34.9	26.3	15.1	58.6	41.4	−0.193	0.273
*rpl6*	492	28.9	32.7	23.4	15.0	61.6	38.4	−0.063	0.217
*rps2*	558	29.2	35.1	20.8	14.9	64.3	35.7	−0.092	0.166
*rps4*	714	30.1	33.1	19.7	17.1	63.2	36.8	−0.047	0.072
*nad1*	969	21.3	39.5	23.7	15.5	60.8	39.2	−0.301	0.211
*tatC*	726	33.3	34.7	12.7	19.3	68.0	32.0	−0.020	−0.207
*rps12*	381	31.2	27.6	23.6	17.6	58.8	41.2	0.063	0.146
*rps7*	600	30.8	33.3	19.0	16.8	64.2	35.8	−0.039	0.060
*nad4L*	303	24.1	43.6	19.5	12.9	67.7	32.3	−0.288	0.204
*rpl14*	387	28.4	29.2	25.3	17.1	57.6	42.4	−0.013	0.195
*rpl5*	516	27.7	39.0	20.0	13.4	66.7	33.3	−0.169	0.198
*atp9*	228	19.7	37.7	27.6	14.9	57.5	42.5	−0.313	0.299
*orf109*	330	40.9	25.5	16.1	17.6	66.4	33.6	0.233	−0.045
*rpl16*	408	37.7	21.6	21.1	19.6	59.3	40.7	0.273	0.036
*rps3*	822	43.3	23.5	16.1	17.2	66.8	33.2	0.297	−0.033
*rps19*	243	37.9	24.7	15.6	21.8	62.6	37.4	0.211	−0.165
*rpl2*	720	36.1	22.4	22.4	19.2	58.5	41.5	0.235	0.077
*rps13*	345	28.4	33.9	21.4	16.2	62.3	37.7	−0.088	0.138
*rps11*	405	32.3	29.4	22.2	16.0	61.7	38.3	0.048	0.161
*cox3*	813	19.7	38.1	26.0	16.2	57.8	42.2	−0.319	0.230
*atp6*	753	20.3	40.9	23.0	15.8	61.2	38.8	−0.336	0.185
*nad2*	1488	21.0	43.9	19.6	15.5	64.9	35.1	−0.353	0.117
*cox1*	1530	20.6	37.5	22.9	19.1	58.0	42.0	−0.291	0.090
*atp8*	261	26.4	42.9	20.3	10.3	69.3	30.7	−0.238	0.325
*nad9*	588	25.3	33.7	24.0	17.0	59.0	41.0	−0.141	0.170
*cob*	1155	22.0	38.8	22.6	16.6	60.8	39.2	−0.276	0.152
*cox2*	846	24.7	35.8	23.3	16.2	60.5	39.5	−0.184	0.180
*nad4*	1443	21.8	40.3	21.6	16.4	62.0	38.0	−0.298	0.139
*nad5*	1992	21.6	39.1	22.4	16.9	60.7	39.3	−0.289	0.139
*nad6*	822	22.6	44.8	18.0	14.6	67.4	32.6	−0.329	0.104
*nad11*	603	25.9	34.0	26.0	14.1	59.9	40.1	−0.136	0.298
*nad3*	360	22.5	42.8	20.0	14.7	65.3	34.7	−0.311	0.152
*rps14*	297	33.7	30.6	20.9	14.8	64.3	35.7	0.047	0.170
*nad7*	1197	26.3	30.5	25.4	17.8	56.8	43.2	−0.074	0.176
Total	23,982	26.1	35.8	21.7	16.5	61.8	38.2	-	-

**Table 4:** Features of the mitogenome PCGs in four Dictyotaceae family species.

PCG	Length (bp)	Amino Acids	Start Codon	Stop Codon	Strand
*rps10*	315 ^a,b^/330 ^c^/291 ^d^	104 ^a,b^/109 ^c^/96 ^d^	ATG	TAG ^a^/TAA ^b,d^/TGA ^c^	H
*rps8*	372	123	ATG	TGA	H
*rpl6*	492	163	ATG	TAA	H
*rps2*	558 ^a^/567 ^b,d^/570 ^c^	185 ^a^/188 ^b,d^/189 ^c^	ATG	TAA	H
*rps4*	714 ^a,b^/711 ^c^/705 ^d^	237 ^a,b^/236 ^c^/234 ^d^	ATG	TAG ^a,b,c^/TAA ^d^	H
*nad1*	969 ^a,b^/975 ^c,d^	322 ^a,b^/324 ^c,d^	ATG	TAA	H
*tatC*	726 ^a^/720 ^b,d^/768 ^c^	241 ^a^/239 ^b,d^/255 ^c^	ATG	TAA ^a,c^/TAG ^b,d^	L
orf *	114 ^b^/117 ^c^	37 ^b^/38 ^c^	TTG ^b^/CTG ^c^	TAA ^b,c^	H ^b,c^
*rps12*	381 ^a,b^/384 ^c^/375 ^d^	126 ^a,b^/127 ^c^/124 ^d^	ATG	TAG ^a,b^/TGA ^c^/TAA ^d^	H
*rps7*	600 ^a^/594 ^b^/597 ^c^/531 ^d^	199 ^a^/197 ^b^/198 ^c^/176 ^d^	ATG	TAA ^a,b,d^/TGA ^c^	H
*nad4L*	303	100	ATG	TAA ^a,d^/TGA ^b,c^	H
*rpl14*	387 ^a,b,c^/381 ^d^	128 ^a,b,c^/126 ^d^	ATG	TGA ^a^/TAA ^b,c,d^	H
*rpl5*	516 ^a,b^/519 ^c^/531 ^d^	171 ^a,b^/172 ^c^/176 ^d^	ATG	TAG ^a^/TAA ^b,c,d^	H
*atp9*	228	75	ATG	TAA	H
orf *	330 ^a^/336 ^b^/366 ^c^	109 ^a^/111 ^b^/121 ^c^	ATG ^a,b,c^	TAA ^a,b,c^	L ^a,b,c^
*rpl16*	408	135	ATG	TGA ^a,b^/TAA ^c,d^	L
*rps3*	822 ^a^/834 ^b^/837 ^c^/777 ^d^	273 ^a^/277 ^b^/278 ^c^/258 ^d^	ATG	TAA	L
*rps19*	243 ^a,b^/258 ^c^/255 ^d^	80 ^a,b^/85 ^c^/84 ^d^	ATG	TAA ^a,b,c^/TGA ^d^	L
*rpl2*	720 ^a,b^/732 ^c^/690 ^d^	239 ^a,b^/243 ^c^/229 ^d^	ATG	TAA	L
*rps13*	345 ^a^/348 ^b,c^/351 ^d^	114 ^a^/115 ^b,c^/116 ^d^	ATG	TAA	H
*rps11*	405 ^a^/417 ^b^/402 ^c^/396 ^d^	134 ^a^/138 ^b^/133 ^c^/131 ^d^	ATG	TAA ^a,c,d^/TAG ^b^	H
*cox3*	813 ^a^/807 ^b,c^/801 ^d^	270 ^a^/268 ^b,c^/266 ^d^	ATG	TAA ^a,d^/TGA ^b,c^	H
*atp6*	753	250	ATG	TAA ^a,b,d^/TGA ^c^	H
*nad2*	1488 ^a,b,c^/1491 ^d^	495 ^a,b,c^/496 ^d^	ATG	TAA	H
*cox1*	1530 ^a,b^/1539 ^c^/1485 ^d^	509 ^a,b^/512 ^c^/494 ^d^	ATG	TAA ^a,b,c^/TAG ^d^	H
*atp8*	261	86	ATG	TGA ^a,b^/TAA ^c,d^	H
*nad9*	588 ^a^/579 ^b^/573 ^c^/582 ^d^	195 ^a^/192 ^b^/190 ^c^/193 ^d^	ATG	TAA ^a,c,d^/TGA ^b^	H
*cob*	1155 ^a,b^/1152 ^c^/1158 ^d^	384 ^a,b^/383 ^c^/385 ^d^	ATG	TGA ^a,b,c^/TAA ^d^	H
*rpl31*	201 ^b^/231 ^c^/207 ^d^	66 ^b^/76 ^c^/68 ^d^	ATG ^b,c,d^	TAG ^b^/TGA ^c^/TAA ^d^	H ^b,c,d^
*cox2*	846 ^a^/885 ^b^/873 ^c^/888 ^d^	281 ^a^/294 ^b^/290 ^c^/295 ^d^	ATG	TAA ^a,c,d^/ TAG ^b^	H
*nad4*	1443 ^a,b^/1437 ^c,d^	480 ^a,b^/478 ^c,d^	ATG	TAA ^a,b,d^/TAG ^c^	H
*nad5*	1992 ^a^/1989 ^b,c^/1998 ^d^	663 ^a^/662 ^b,c^/665 ^d^	ATG	TAA	H
orf *	165 ^b^/156 ^c^	54 ^b^/51 ^c^	ATG ^b,c^	TGA ^b^/TAG ^c^	H ^b,c^
*nad6*	822 ^a,b^/834 ^c^/792 ^d^	273 ^a,b^/277 ^c^/262 ^d^	ATG	TAA	H
*nad11*	603	200	ATG	TGA	H
*nad3*	360 ^a,b,d^/363 ^c^	119 ^a,b,d^/200 ^c^	ATG	TAA ^a,b,d^/TGA ^c^	H
*rps14*	297	98	GTG ^a,b^/ATG ^c,d^	TAA	H
*nad7*	1197 ^a,b,c^/1200 ^d^	398 ^a,b,c^/399 ^d^	ATG	TAA	H

^a^ *D*. *coriacea* (PV670818), ^b^ *D*. *dichotoma* (AY500368), ^c^ *D*. *divaricata* (MG940856), ^d^ *D*. *propagulifera* (ON552984), * ORF indicates unidentified frame.

## Data Availability

Data associated with this study has been deposited at NCBI under the accession number PV670818 (https://www.ncbi.nlm.nih.gov/nuccore/PV670818). All data generated or analyzed during this study are included in this article and its Supplementary Information files.

## Supplementary Materials

The following supporting information can be downloaded at: https://www.mdpi.com/article/10.3390/life15101605/s1, Figure S1: A specimen image of *Dictyota coriacea* (Class Phaeophyceae) collected from the coast of Busan, South Korea. This marine brown macroalga is 20 to 30 cm tall, thin, ribbon-shaped, branches out into two branches several times to form a wide fan shape, and branches twisted once or twice; Table S1: Summary of *Dictyota coriacea* mitogenome data produced/stats during de novo assembly analysis in Illumina platform using NOVOPlasty v4.2.1 assembly method; Table S2. List of Phaeophyceae species mitogenome were used in this study; Table S3: Annotation of mitochondrial functional genes of *Dictyota coriacea*; Table S4: Relative Synonymous Codon Usage (RSCU) values of complete protein-coding genes in the mitogenome of Dictyotaceae species; Table S5: Mitochondrial rRNA in Dictyotaceae species; Table S6: Mitochondrial tRNA in Dictyotaceae species.
