# Supplementary material for: The First Complete Mitogenome Characterization of Brown Alga Dictyota coriacea (Phaeophyceae, Heterokontophyta) and Its Phylogenetic Analysis
Source: Life (Basel). 2025 Oct 15;15(10):1605. doi: 10.3390/life15101605 (PMC12565469; doi:10.3390/life15101605)
Supplement: Supplementary file 1 [file life-15-01605-s001.zip › life-3882285-supplementary.pdf]

<u>**SUPPLEMENTARY DATA**</u>

The First Complete Mitogenome Characterization of Brown Alga *Dictyota coriacea* (Phaeophyceae, Heterokontophyta) and Its Phylogenetic Analysis

<u>**Figure:**</u>

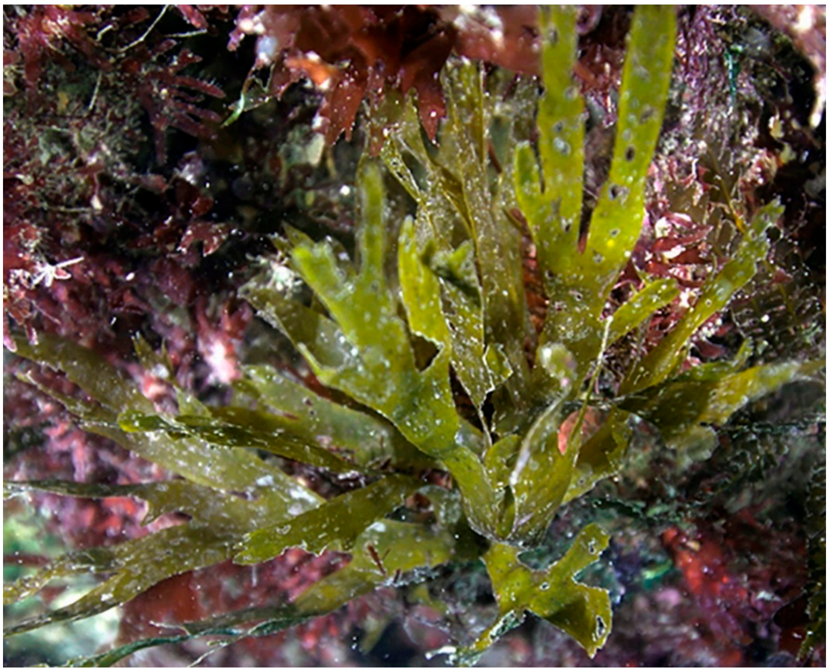

Figure S1. A specimen image of *Dictyota coriacea* (Class Phaeophyceae, Ochrophyta) collected from the coast of Busan, South Korea. This marine brown macroalga is 20 to 30 cm tall, thin, ribbon-shaped, branches out into two branches several times to form a wide fan shape, and branches twisted once or twice.

<u>**Tables:**</u>

Table S1. Summary of *Dictyota coriacea* mitogenome data produced/ stats during de novo assembly analysis in Illumina platform using NOVOPlasty v4.2.1 assembly method.

Library	Total read bases	Total reads	GC (%)	Q20 (%)	Q30 (%)	Mapped reads	Coverage (%)	Depth	Insert size (std.)
Raw data stats	2,565,862,064	16,992,464	40.66	97.13	92.38	-	-	-	-
Filtered data stats	2,263,851,265	15,017,576	40.69	98.81	95.09	-	-	-	-
Self-mapping stats	-	15,017,576	-	-	-	32,224	100.00	54.65	344.08 (130.77)

(Coverage %: The percentage of mapped sites (>= 1x). Depth: Average mapping depth. Insert size (std.): The length between adapters and the standard deviation of predicted length.)

Table S2. List of Phaeophyceae species mitogenome were used in this study.

Order	Family	Name	NCBI No.	Length (bp)	A+T (%)
Cutleriales	Cutleriaceae	*Cutleria multifida*	ON552982	37,394	64.0
Desmarestiales	Desmarestiaceae	*Desmarestia viridis*	AY500367	39,049	63.4
Dictyotales	Dictyotaceae	*Dictyota coriacea*	PV670818	31,573	61.2
		Dictyota dichotoma	AY500368	31,617	63.5
		Dictyopteris divaricata	MG940856	32,021	61.7
		Dictyotopsis propagulifera	ON552984	30,995	73.2
Ectocarpales	Acinetosporaceae	*Pilayella littoralis*	AJ277126	58,507	62.0
	Scytosiphonaceae	*Scytosiphon lomentaria*	KJ995702	36,918	65.9
		Colpomenia peregrina	KM244739	36,025	68.0
		Endarachne binghamiae	MF374731	37,460	65.6
		Petalonia fascia	KJ957769	38,053	66.5
		Hapterophycus canaliculatus	ON552990	37,846	66.4
	Ectocarpaceae	*Ectocarpus siliculosus*	FP885846	37,187	66.5
	Chordariaceae	*Cladosiphon okamuranus*	MG488292	38,419	65.7
		Pleurocladia lacustris	KU164873	37,814	67.1
Ishigeales	Ishigeaceae	*Ishige okamurae*	ON552991	35,511	64.7
Fucales	Fucaceae	*Fucus vesiculosus*	AY494079	36,392	65.6
		Silvetia siliquosa	MW485976	36,036	66.3
	Sargassaceae	*Coccophora langsdorfii*	KU255794	35,660	63.6
		Turbinaria ornata	KM501562	34,981	64.2
		Myagropsis myagroides	MT920491	34,845	64.0
		Sargassum horneri	KJ938300	34,606	63.8
Laminariales	Agaraceae	*Dictyoneurum californicum*	MZ156053	37,840	66.8
		Thalassiophyllum clathrus	MZ156067	37,643	65.5
		Agarum clathratum	ON552977	37,571	65.6
		Costaria costata	KF384641	37,461	65.1
	Akkesiphycaceae	*Akkesiphycus lubricus*	MZ156045	39,330	62.4
	Alariaceae	*Pleurophycus gardneri*	MZ156059	39,142	65.8
		Alaria esculenta	OK148994	38,857	65.2
		Pterygophora californica	MZ156061	38,715	66.1
		Lessoniopsis littoralis	MZ156066	38,444	66.6
		Undaria pinnatifida	KF319031	37,402	67.5
	Chordaceae	*Chorda filum*	PP316522	41,946	62.5
	Laminariaceae	*Laminaria digitata*	AJ344328	38,007	64.9
		Saccharina japonica	AP011493	37,657	64.7
		Macrocystis pyrifera	ON227496	37,326	68.2
		Pelagophycus porra	MZ156058	37,465	67.7
		Postelsia palmaeformis	MZ156060	37,455	64.4
		Nereocystis luetkeana	MH392199	37,399	64.7
		Hedophyllum nigripes	MZ156046	52,071	63.2
		Arthrothamnus bifidus	MZ156049	38,790	64.6
		Cymathaere triplicata	MZ156051	37,998	63.9
	Lessoniaceae	*Ecklonia arborea*	MZ156048	39,843	67.1
		Lessonia spicata	MZ156056	37,097	67.4
		Eisenia bicyclis	ON552988	37,539	68.1
		Egregia menziesii	ON552987	37,187	69.1

Order	Family	Name	NCBI No.	Length (bp)	A+T (%)
	Pseudochordaceae	*Pseudochorda nagaii*	MZ156063	40,990	61.5
Ralfsiales	Ralfsiaceae	*Analipus japonicus*	MZ156065	38,173	59.2
Scytothamnales	Scytothamnus	*Scytothamnus australis*	ON552995	37,778	59.9
Sphacelariales	Choristocarpaceae	*Choristocarpus tenellus*	ON552980	32,351	69.4
Sporochnales	Sporochnaceae	*Sporochnus bolleanus*	ON552996	36,940	61.6
Syringodermatales	Syringodermataceae	*Syringoderma abyssicola*	ON552997	36,719	60.2
Tilopteridales	Halosiphonaceae	*Halosiphon tomentosus*	ON552989	39,326	60.2
Out group species	-	*Schizocladia ischiensis*	MT259947	-	-

Table S3. Annotation of mitochondrial functional genes of *Dictyota coriacea*.

Type	Group of Gene		Name of Gene
RNAs	rRNA	The large subunit of a ribosome	*rnl*
		The small subunit of a ribosome	*rrn5*, *rns*
	tRNA	Transfer RNA genes	*trnA*, *trnC*, *trnD*, *trnE*, *trnF*, *trnG*, *trnH*, *trnI*, *trnK*, *trnL*1, *trnL*2, *trnL*3, *trnM*1, *trnM*2, *trnM*3, *trnN*1, *trnN*2, *trnP*, *trnQ*, *trnR*, *trnS*1, *trnS*2, *trnV*, *trnW*, *trnY*
PCGs	Respiratory chain subunits	NADH dehydrogenase subunits	*nad1*, *nad2*, *nad3*, *nad4*, *nad4L*, *nad5*, *nad6*, *nad7*, *nad9*, *nad11*
		Apocytochrome b	*cob*
		Cytochrome c oxidase subunit	*cox1*, *cox2*, *cox3*
	ATP synthase subunits		*atp6*, *atp8*, *atp9*
	Ribosomal proteins	Small subunit (SSU)	*rps2*, *rps3*, *rps4*, *rps7*, *rps8*, *rps10*, *rps11*, *rps12*, *rps13*, *rps14*, *rps19*,
		Large subunit (LSU)	*rpl2*, *rpl6*, *rpl5*, *rpl14*, *rpl16*
	Independent protein translocase		*tatC*

Table S4. Relative Synonymous Codon Usage (RSCU) values of complete protein-coding genes in the mitogenome of Dictyotaceae species.

Codon	*Dictyota coriacea* (PV670818)		*Dictyota dichotoma* (AY500368)		*Dictyopteris divaricata* (MG940856)		*Dictyotopsis propagulifera* (ON552984)	
	Count	RSCU	Count	RSCU	Count	RSCU	Count	RSCU
UUU(F)	512	1.66	550	1.74	550	1.75	690	1.95
UUC(F)	106	0.34	81	0.26	80	0.25	16	0.05
UUA(L)	465	2.68	499	2.75	411	2.31	763	4.38
UUG(L)	260	1.15	279	1.54	279	1.57	102	0.59
CUU(L)	115	0.66	137	0.76	191	1.07	140	0.80
CUC(L)	32	0.18	31	0.17	31	0.17	1	0.01
CUA(L)	98	0.57	90	0.50	100	0.56	33	0.19
CUG(L)	70	0.40	52	0.29	55	0.31	7	0.04
AUU(I)	276	1.36	340	1.53	332	1.57	516	1.79
AUC(I)	92	0.45	75	0.34	76	0.36	356	0.12
AUA(I)	239	1.18	250	1.13	228	1.08	315	1.09
AUG(M)	201	1.00	195	1.00	203	1.00	171	1.00
GUU(V)	316	2.02	353	2.18	363	2.25	269	2.45
GUC(V)	65	0.42	59	0.36	61	0.38	9	0.08
GUA(V)	151	0.96	152	0.94	115	0.71	132	1.20
GUG(V)	94	0.60	84	0.52	105	0.65	29	0.26
UCU(S)	184	1.75	189	1.79	202	1.83	248	2.41
UCC(S)	69	0.66	53	0.50	74	0.67	10	0.10
UCA(S)	88	0.84	103	0.98	107	0.97	133	1.29
UCG(S)	90	0.86	83	0.79	66	0.60	29	0.28
CCU(P)	82	1.08	83	1.14	99	1.25	146	2.32
CCC(P)	89	1.17	58	0.80	78	0.99	7	0.11
CCA(P)	63	0.83	82	1.13	78	0.99	82	1.30
CCG(P)	71	0.93	68	0.93	61	0.77	17	0.27
ACU(T)	108	1.16	123	1.33	120	1.21	154	1.81
ACC(T)	72	0.77	76	0.82	90	0.91	11	0.13
ACA(T)	98	1.05	108	1.16	130	1.31	164	1.92
ACG(T)	95	1.02	64	0.69	56	0.57	12	0.14
GCU(A)	154	1.46	179	1.81	178	1.67	196	2.17
GCC(A)	88	0.84	72	0.73	89	0.84	21	0.23
GCA(A)	79	0.75	82	0.83	96	0.90	123	1.36
GCG(A)	100	0.95	63	0.64	63	0.59	21	0.23
UAU(Y)	227	1.33	267	1.50	232	1.34	324	1.79
UAC(Y)	114	0.67	90	0.50	115	0.66	39	0.21
UAA(*)	0	0	0	0	0	0	0	0
UAG(*)	0	0	0	0	0	0	0	0
CAU(H)	102	1.24	106	1.33	109	1.35	149	1.82
CAC(H)	62	0.76	53	0.67	53	0.65	15	0.18
CAA(Q)	115	1.26	149	1.51	154	1.48	170	1.74
CAG(Q)	67	0.74	49	0.49	54	0.52	25	0.26
AAU(N)	233	1.39	270	1.53	208	1.31	373	1.74
AAC(N)	103	0.61	84	0.47	109	0.69	56	0.26
AAA(K)	314	1.48	340	1.50	352	1.57	540	1.90
AAG(K)	111	0.52	112	0.50	97	0.43	29	0.10

GAU(D)	164	1.44	175	1.55	173	1.45	173	1.81
GAC(D)	63	0.56	51	0.45	65	0.55	18	0.19
GAA(E)	120	1.00	155	1.25	124	1.04	195	1.82
GAG(E)	121	1.00	93	0.75	114	0.96	19	0.18
UGU(C)	101	1.49	112	1.61	88	1.47	102	1.76
UGC(C)	35	0.51	27	0.39	32	0.53	14	0.24
UGA(*)	0	0	0	0	0	0	0	0
UGG(W)	137	1.00	138	1.00	137	1.00	128	1.00
CGU(R)	108	1.73	129	2.13	101	1.58	101	2.62
CGC(R)	46	0.74	33	0.54	45	0.70	10	0.26
CGA(R)	48	0.77	53	0.87	40	0.63	59	1.53
CGG(R)	59	0.95	38	0.63	46	0.72	8	0.21
AGU(S)	132	1.26	159	1.51	155	1.40	179	1.74
AGC(S)	68	0.65	45	0.43	60	0.54	19	0.18
AGA(R)	70	1.12	68	1.12	73	1.14	52	1.35
AGG(R)	43	0.69	43	0.71	78	0.12	1	0.03
GGU(G)	248	1.73	298	2.05	283	1.89	230	2.04
GGC(G)	96	0.67	59	0.41	76	0.51	23	0.20
GGA(G)	76	0.53	85	0.59	74	0.49	175	1.55
GGG(G)	154	1.07	139	0.96	167	1.11	24	0.21
Total	7959	-	8133	-	8181	-	7852	-

Note: * = the termination codon, A = Ala, F = Phe, C = Cys, D = Asp, N = Asn, E = Glu, Q = Gln, G = Gly, H = His, L = Leu, I = Ile, K = Lys, M = Met, P = Pro, R = Arg, S = Ser, T = Thr, V = Val, W = Trp, Y = Tyr

Table S5. Mitochondrial rRNA in Dictyotaceae species.

**rRNA**	***Dictyota coriacea***	***Dictyota dichotoma***	***Dictyopteris divaricata***	***Dictyotopsis propagulifera***
rrn5	113 bp	133 bp	137 bp	138 bp
rns	1525 bp	1557 bp	1529 bp	1512 bp
rnl	2668 bp	2642 bp	2663 bp	2662 bp

Table S6. Mitochondrial tRNA in Dictyotaceae species.

tRNA (codon)	*Dictyota coriacea*	*Dictyota dichotoma*	*Dictyopteris divaricata*	*Dictyotopsis propagulifera*
trnA (TGC)	1	1	1	1
trnC (GCA)	1	1	1	1
trnD (GTC)	1	1	2	1
trnE (TTC)	1	1	0	1
trnF (GAA)	1	1	1	1
trnG (GCC)	1	1	1	1
trnH (GTG)	1	1	1	1
trnI (GAT)	1	2	1	1
trnK (TTT)	1	2	1	1
trnL (CAA)	1	1	1	1
trnL (TAA)	1	1	1	1
trnL (TAG)	1	1	1	1
trnM (CAT)	3	2	3	2
trnN (GTT)	2	1	1	1
trnP (TGG)	1	1	1	1
trnQ (TTG)	1	1	1	1
trnR (TCT)	1	1	1	1
trnS (GCT)	1	1	1	1
trnS (TGA)	1	1	1	1
trnV (TAC)	1	1	1	1
trnW (TCA)	1	1	1	1
trnY (GTA)	1	1	1	1
**Total tRNA**	25	25	24	23
